# WAP four-disulfide core domain protein 2 gene(*WFDC2*) is a target of estrogen in ovarian cancer cells

**DOI:** 10.1186/s13048-015-0210-y

**Published:** 2016-02-29

**Authors:** Yao Chen, Suihai Wang, Tiancai Liu, Yingsong Wu, Ji-Liang Li, Ming Li

**Affiliations:** School of Biotechnology, Southern Medical University, 1023 Shatainan Road, Guangzhou, 510515 China; Molecular Oncology Laboratories, Department of Oncology, Weatherall Institute of Molecular Medicine, University of Oxford, Oxford, OX3 9DS UK

**Keywords:** *WFDC2*, Ovarian cancer, Estrogen, Cell proliferation, Apoptosis

## Abstract

**Background:**

WAP four-disulfide core domain protein 2 (*WFDC2*) shows a tumor-restricted upregulated pattern of expression in ovarian cancer.

**Methods:**

We investigated the role of estradiol (E2) on cell growth in estrogen-sensitive or estrogen-insensitive ovarian cancer cell lines. Real-time (RT)-PCR and western blotting were used to examine the expression of *WFDC2* at RNA and protein levels. Growth traits of cells transfected with *WFDC2*-shRNA or blank control were assessed using MMT arrays. Cell apoptosis was analyzed using annexin V-FITC/PI and flow cytometry. Estrogen receptor expression was evaluated using RT-PCR and flow cytometry. Apoptosis-related proteins induced by E2 directly and indirectly were determined using an antibody array comparing cells transfected with *WFDC2*- shRNA or a blank control.

**Results:**

High-dose (625 ng/ml) E2 increased the expression of *WFDC2* in HO8910 cells at both the mRNA and protein levels. However, E2 had no effect on *WFDC2* expression in estrogen-insensitive SKOV3 cells. Of interest, knockdown of *WFDC2* enabled a considerable estrogen response in SKOV3 cells in terms of proliferation, similar to estrogen-responsive HO8910 cells. This transformation of SKOV3 cells into an estrogen-responsive phenotype was accompanied by upregulation of estrogen receptor beta (ERß) and an effect on cell apoptosis under E2 treatment by regulating genes related to cell proliferation and apoptosis.

**Conclusions:**

We postulate that increased *WFDC2* expression plays an important role in altering the estrogen pathway in ovarian cancer, and the identification of *WFDC2* as a new player in endocrine-related cancer encourages further studies on the significance of this gene in cancer development and therapy.

**Electronic supplementary material:**

The online version of this article (doi:10.1186/s13048-015-0210-y) contains supplementary material, which is available to authorized users.

## Background

Ovarian cancer is one of the most common cancers among women and the leading cause of death from gynecological malignancies in the world [1, 2]. The underlying causes of ovarian cancer are poorly understood and largely untested, but estrogen, as a major steroidal product of the ovary, has been shown to be associated with increased ovarian cancer risk in estrogen receptor (ER)-expressing cells [3–5]. Hormone replacement therapy (HRT) has been widely used in women with estrogen-withdrawal syndromes. Estrogen has been associated with an increased ovarian cancer risk and it can promote tumor growth and cell proliferation in ER-expressing cell lines. The main biological functions of estrogen are manifested through transcriptional activation of the ligand-dependent ERs, ERα and ERβ [3, 6]. More than two-thirds of ovarian cancer patients are positive for ERα [6–8]. The activation of ER results in an altered expression of its direct transcriptional targets, thereby affecting a series of downstream secondary biological activities. However, the regulatory effects of estrogen on the behavior of ovarian tumor cells involves a complex signaling network and the underlying mechanisms are still not fully understood. Therefore, identification of novel targets regulated by estrogen will be very important to clarify the specific impact of estrogen on ovarian tumor growth and facilitate the development of new diagnostic and therapeutic markers [4, 5, 9].

In recent years, a number of studies have shown that numerous estrogen-responsive genes, including insulin-like growth factor binding protein (IGFBP) family members (*IGFBP3*, *IGFBP4* and *IGFBP5*), trefoil factor (*TFF*) family members (*TFF1* and *TFF3*), and TNF receptor-associated protein 1 (*TRAP1*) among others, affect the growth and development of ovarian cancer [10, 11]. Genomic analysis of ovarian cancer identified gene amplification of the *WFDC2* gene (that encodes the human epididymis protein 4 (*HE4*)) and the whey acidic protein gene loci in a large proportion of epithelial ovarian cancers [12, 13]. HE4 exhibits a tumor-restricted, upregulated pattern of expression in ovarian cancer, making it a potential marker [12, 14, 15]. The previous work has shown a direct linkage between *HE4* expression and cell proliferation; however the molecular mechanisms are still unclear [12, 16]. To date, the majority of studies have focused on the potential value of HE4 as a diagnostic using various serologic tests, but very little attention has been paid to the role of HE4 in tumor development of ovarian cancer [12, 14, 17].

The *WFDC2* gene is located on human chromosome 20q12-13.1, a region that includes 14 genes that encode proteins with a WAP-type four-disulfide core (WFDC) domain [14, 17]. Two of the best-studied members of the WAP gene family are *SLPI* and *PI3* (that encodes for elafin), both having antiproteinase activity. They are co-expressed with *WFDC2* and involved in cancer development or progression in various carcinomas affected by sex hormones [9, 14, 18]. So we could not help to speculate that WFDC2 might also play some role in the estrogen-sensitive ovarian cancers.

As a cancer-specific gene, several hormone-response elements were found within the *WFDC2* promoter, including an estrogen response element (ERE) and RORA, which may be attributed to HE4 upregulation in ovarian cancer and ovarian cancer specificity [19]. The amount of HE4 in blood samples was significantly different between follicular (FP) and ovulatory (OP) phases of the hormonal cycle, being lower in the FP than in the OP [20]. The menstrual cycle phase-dependent variability indicated that *WFDC2* expression might be affected by the menstrual cycle of women. These results suggested that *WFDC2* might be an estrogen response gene, and play important roles in the cell proliferation and malignant transformation of ovarian cancer.

In this study, we investigated the regulatory effects of estrogen and estrogen antagonist on *WFDC2* gene expression in estrogen sensitive HO8910 cells and estrogen insensitive SKOV3 cells, with the aim to determine whether *WFDC2* is an estrogen-responsive gene. And then, we transfected these cells with short hairpin RNA (shRNA) against *WFDC2*, and investigated the effect of *WFDC2* silencing on cell proliferation, its interaction with ER and its effect on ER-mediated signaling.

## Methods

### Cells and treatments

The cell bank of the Chinese Academy of Sciences (Shanghai, China) supplied the human ovarian cancer cell lines, HO8910 and SKOV3 (American Type Culture Collection (ATCC), Manassas, VA, USA). Cells were maintained in minimal essential medium supplemented with 10 % (*v/v*) fetal bovine serum (FBS) at 37 °C in an atmosphere of 95 % air, 5 % CO_2_. The ligand 17β-estradiol (E2) and the selective ER modulator (SERM), tamoxifen (TAM), were purchased from Sigma-Aldrich (St Louis, MO, USA). Before the cells were treated with the ligands, the medium was replaced with minimal essential medium supplemented with 0.5 % FBS. Cells were treated with different concentrations of E2 (5, 25, 125, 625 and 1250 ng/ml), and TAM (100 ng/ml), for 24 h prior to quantitative real-time PCR (QPCR) and 48 h prior to western blotting and protein array analysis.

### RNA extraction and QPCR

Total RNA was isolated following the manufacturer’s instructions (PrimeScript 1st Strand cDNA Synthesis Kit, TAKARA). Reverse transcription was also performed following the manufacturer’s instructions in a total volume of 20 μl using an oligo-dT primer and 1 μg of total RNA. Each primer set was designed using Primer Express software v3.0 to flank an intron to prevent the amplification of genomic DNA. β-actin was used to evaluate the efficiency and variability of the reverse transcription step. CDNA samples (0.1 μg) were amplified using the SYBR Green PCR Master Mix (TAKARA) under conditions recommended by the manufacturer: (a) pre-incubation at 95 °C for 30 s; (b) 40 PCR cycles of 95 °C for 5 s, 55 °C for 30 s, and 72 °C for 34 s. Samples were assayed in duplicate using the ABI Prism 7500 detection system (Perkin Elmer Applied Biosystems). The relative quantification number was then calculated by subtracting the average CT from the corresponding average CT for β-actin.

### Western blotting

Total protein was extracted using sonication in radio-immunoprecipitation assay (RIPA) buffer (50 mM Tris–HCl pH 7.5, 150 mM NaCl, 5 mM EDTA, 0.5 % Nonidet P-40, 5 mM dithiothreitol, 10 mM NaF, and protease inhibitor cocktail). One hundred micrograms of denatured protein was separated on an SDS-polyacrylamide gel and transferred to a Hybond membrane (Amersham, Germany), which was then blocked overnight in 5 % skim milk in Tris-buffered saline with Tween 20 (TTBS, 10 mM Tris–HCl, 150 mM NaCl, 0.1 % Tween 20). For immunoblotting, the membrane was incubated for 15 min with the *WFDC2* antibody. The membrane was rinsed with TTBS and incubated with anti-rabbit IgG conjugated to horseradish peroxidase (DAKO, USA, 1:1000) for 15 min. All incubations were performed in a microwave oven to allow intermittent irradiation. Bands were visualized on an ImageQuant LAS4010 (GE Healthcare Life Science, USA) using ECL-Plus detection reagents (Santa Cruz, USA). Densitometric quantification of protein bands with GAPDH as an internal control was performed using Image J (NIH, USA).

### Gene silencing

The *WFDC2*-specific shRNA sequence (5′-GCTCTCTGCCCAATGATAAGG-3′) (based on the Gene Bank Accession No. NM_0006103.3) and its control sequence (5′-GTTCTCCGAACGTGTCACGT-3′) were chemically synthesized and cloned into the pGLV-U6-GFP vector by Shanghai GenePharma Co. Ltd (Camsonne et al.). Lentiviruses, purchased from the same company, were transduced into the HO8910 cell line according to the manufacturer’s instructions. For stable silencing of *WFDC2*, the transduced HO8910 cell line, named HO8910-209, was selected using puromycin. Puromycin-resistant colonies were then picked, expanded and analyzed separately.

### Cell proliferation assays

The in vitro proliferation assay was performed using the 3-(4, 5-dimethylthiazol-2-yl)-2, 5-diphenyltetrazolium bromide (MTT) assay following the manufacturer’s instructions (Sigma). Briefly, 500 cells per well were plated in 96-well plates in triplicate. After a 24 h incubation, cells were serum-starved for 24 h and then treated with different concentrations of estrogen (0, 5, 25, 125, 625 and 1250 ng/ml) or TAM for 6 days. At the indicated time, cell proliferation was determined by measurement of the absorbance values using the MTT assay method, and cell growth curves were then plotted.

### Flow cytometry

Cells were trypsinized and washed three times with phosphate-buffered saline (PBS). Cells were then digested with 1 % RNase at 37 °C for 30 min and stained with annexin V-FITC for 30 min, followed by staining with propidium iodide (PI, Sigma, Shanghai, China) for 5 min at 4 °C for cell apoptotic analysis. The results were analyzed using WinMDI software with 10,000 events collected for each sample. Cell suspensions were also incubated with antibodies to ERα and ERß (1 μg/1 × 10^6^ cells) for 30 min at 37 °C. Cells were then washed three times with PBS, and incubated with PE and an FITC-labeled secondary antibody for 30 min at 37 °C. Cell suspensions were washed three times with PBS. Expression of ERα and ERß per 10000 cells was determined using flow cytometry.

### Annexin V-FITC/PI staining

Apoptosis was evaluated using the Annexin V-FITC/PI Apoptosis Detection kit (BestBio, Shanghai, China) using fluorescence microscopy according to the manufacturer’s instructions. Briefly, cells were grown onto a cover slide and incubated with E2 (625 ng/ml) for 24 h. The adherent cells were washed twice with ice-cold PBS and stained with annexin V-FITC and PI for 15 min and examined under a light microscope equipped with appropriate filters. Apoptotic cells stained with annexin V-FITC showed green fluorescence, and necrotic cells stained with both annexin V-FITC and PI produced red fluorescence as well as green fluorescence.

### Human antibody array for apoptotic-related proteins

Apoptotic-related proteins induced by E2 directly and indirectly were determined using the RayBio® Label-Based Human Antibody Array kit (RayBio® Human Apoptosis Antibody array) (RayBiotech, Norcross, GA). Densitometric analysis was performed on a Kodak ImageStation 4000 M (Eastman Kodak Company, Rochester, NY) with background subtraction from spot edges following the manufacturer’s instructions. Spot data were normalized to a positive control spot on each array.

### Statistics

Microsoft Office Excel 2007 and the statistical software SPSS13.0 were used in data processing and the *t*-test was used in analyzing significance. P values < 0.05 were considered statistically significant. Data were expressed as the mean ± SD from at least three independent experiments.

## Results

### High-dose E2 induces expression of *WFDC2* in HO8910 cells

**Fig. 1:**
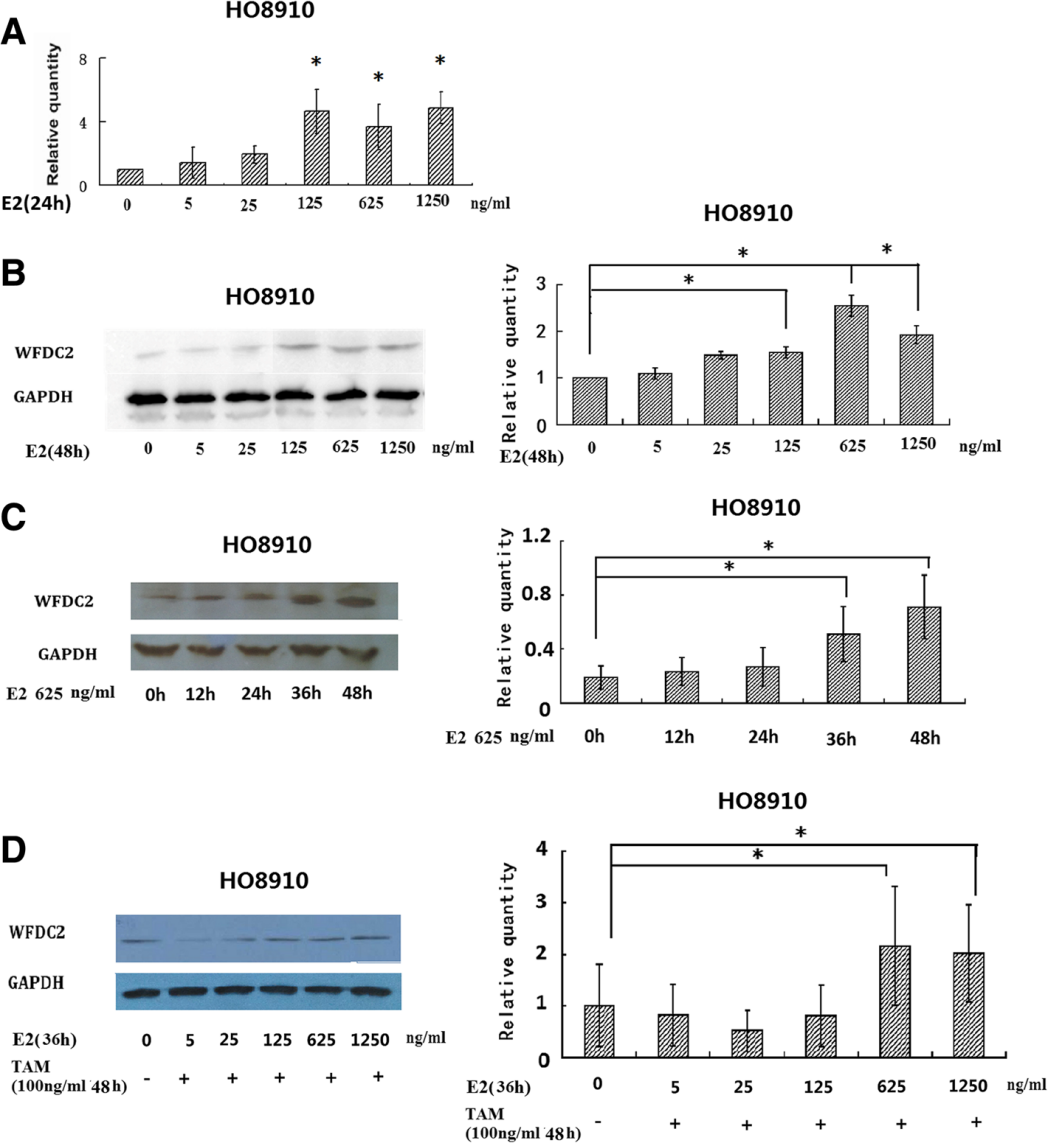
E2 induces expression of *WFDC2* in HO8910 cells. **a** Real-time RT-PCR analysis of the expression of *WFDC2* and GAPDH in HO8910 cells after stimulation with E2 at different concentrations for 48 h (*n* = 3). The relative amounts of *WFDC2* protein were determined using densitometry and normalized to GAPDH. *P <0.05. **b** Western blot analysis of the expression of *WFDC2* and GAPDH in HO8910 cells after stimulation with E2 at different concentrations for 48 h (*n* = 3). The relative amounts of *WFDC2* protein were determined using densitometry and normalized to GAPDH. *P < 0.05 versus the solvent control. **c** Western blot analysis of the expression of *WFDC2* and GAPDH in HO8910 cells after stimulation with 625 ng/ml E2 at different time points. *n* = 3, *P < 0.05 versus 0 h. **d** Western blot analysis of the expression of *WFDC2* and GAPDH in HO8910 cells incubated with TAM (100 ng/ml) for 8 h and then incubated with E2 (625 ng/ml) for 36 h. *n* = 3, *P < 0.05 versus the control

To determine if HE4/*WFDC2* is a downstream target of E2 signaling pathways, we induced the expression of the *WFDC2* gene by adding E2 into the culture of HO8910 cells using a range of concentrations (0, 5, 25, 125, 625 and 1250 ng/ml). The results indicated that the *WFDC2* gene was upregulated only when cells were treated with a high dose of E2. The expression of *WFDC2* at both mRNA and protein levels was increased with E2 from 125 to 1250 ng/ml as detected using QPCR (*P* < 0.05) (Fig. 1a) and western blotting (*P* < 0.05) (Fig. 1b). At a concentration of 625 ng/ml, E2 increased the protein level of *WFDC2* by 2.54-fold. The effect of E2 on *WFDC2* expression was not dose-dependent (Fig. 1c). After 24 h treatment, expression of *WFDC2* was observed to be upregulated and the upregulation was sustained for over 48 h (Fig. 1c).

We next determined whether the ER signaling pathway regulated *WFDC2* mRNA expression. SKOV3 ovarian carcinoma cells have functional ERs but are insensitive to estrogen [21]. We used SKOV3 cells to prove whether *WFDC2* was induced by estrogen. A basal level of *WFDC2* was detected in SKOV3 cells but protein expression was not induced by E2 (data not shown). HO8910 cells were incubated with TAM (100 ng/ml) for 8 h and then with E2 (0, 5, 25, 125, 625 and 1250 ng/ml) for a further 36 h. Cells were then harvested and expression of *WFDC2* analyzed. Amounts of *WFDC2* were increased by adding E2 to HO8910 cells and not affected by adding TAM (Fig. 1d).

These results indicated that *WFDC2* was a downstream target of E2 but that regulation might not be through the ERα pathway.

### Knockdown of *WFDC2* expression reduces cell proliferation driven by estrogen

The dependence of *WFDC2* expression on estrogen activity led us to study whether the proliferative growth of ovarian cancer cells induced by E2 was mediated by *WFDC2*. The sequence 209 shRNA, which was shown to effectively knock down the expression of *WFDC2* in SKOV3 cells [12], was inserted into the retroviral vector and transduced into estrogen-sensitive HO8910 cells to produce the stable *WFDC2* knockdown cell line (HO8910-209) (Additional file 1: Figure S1). Therefore, we compared the growth of HO8910-209 or SKOV3-209 cells with the negative control clone of each cell line to determine the role of *WFDC2* both in estrogen-sensitive and estrogen-insensitive ovarian cancer cells.

**Fig. 2:**
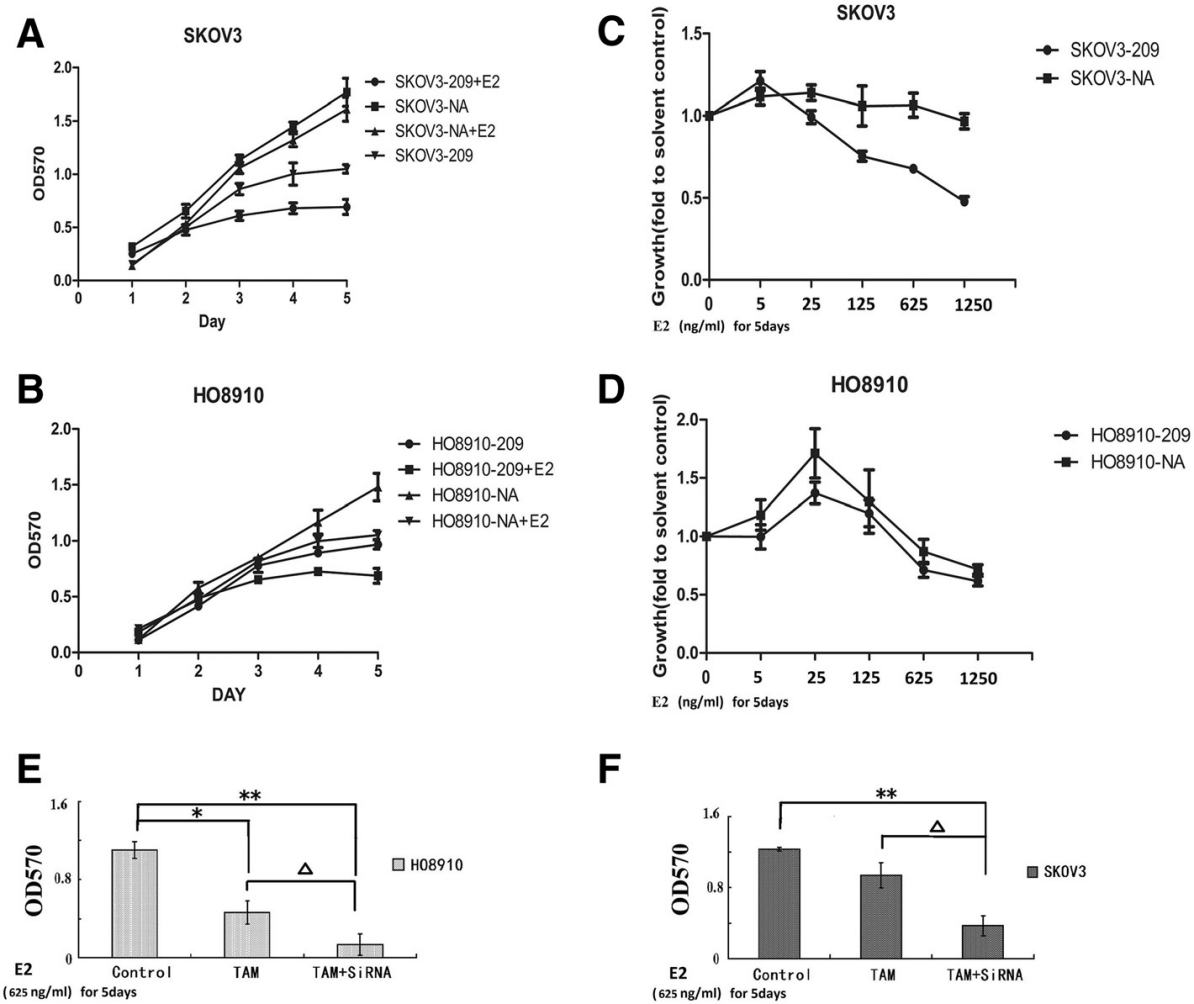
Knockdown of *WFDC2* expression reduces cell proliferation and enables an E2 response in SK-OV-3 ovarian cancer cells. HO8910 (**a**) or SKOV-3 (**b**) cells were treated with or without E2 (625 ng/ml) and knockdown of *WFDC2* reduced the viable cell number compared with the negative control. *n* = 3 *P < 0.05; **P < 0.01. E2 response of HO8910 (**c**) and SKOV3 (**d**) cells. Knockdown of *WFDC2* enabled an E2 response in SK-OV-3 ovarian cancer cells. HO8910 (**e**) or SKOV-3 (**f**) cells were treated with E2 (625 ng/ml) alone or in combination with TAM (100 ng/ml), and knockdown of *WFDC2* increased the inhibition efficiency in ovarian cancer cells by adding TAM compared with the control; *P < 0.05, **P < 0.01 for TAM versus the control. ∆*P* < 0.05, verus cells treated with TAM without ShRNA interference

Combined with previous results, knockdown of the expression of *WFDC2* dramatically decreased the cell proliferation of both HO8910-209 and SKOV3-209 cells (Fig. 2a). Additionally, we performed growth assays with high-dose E2 (625 ng/ml) to analyze the effect of *WFDC2* knockdown on E2-triggered proliferation. Addition of E2 further decreased proliferation of HO8910-209 cells and the relative estrogen response of this cell line was not changed significantly by *WFDC2* knockdown (Fig. 2b). In contrast, addition of high-dose E2 had very little effect on cell proliferation of estrogen-unresponsive SKOV3 cells, while the growth of SKOV3-209 cells was inhibited significantly by high-dose E2 compared with the SKOV3-NA cells. These results indicated that *WFDC2* knockdown might transform the estrogen-unresponsive SKOV3 cells from a hormone-independent to an estrogen-responsive phenotype (Fig. 2b). This transformation of ovarian cancer cells from a hormone-independent to an estrogen-responsive phenotype using knockdown of a single gene was further validated in proliferation experiments combining E2 with the SERM, TAM [22].

In estrogen-sensitive HO8910 cells, treatment with TAM resulted in the expected anti-proliferative effect on this cell line, which was also enhanced by *WFDC2* knockdown (Fig. 2c). Compared with HO8910 cells, treatment with TAM (100 ng/ml) did not affect the growth of SKOV3 cells significantly. While knockdown of *WFDC2* in this cell line enabled a strong inhibitory effect of TAM on E2-triggered growth of ~65 % after 5 days of treatment (Fig. 2c), this drug did not significantly affect the growth of negative control SKOV3 cells.

Thus, the results indicated that knockdown of *WFDC2* not only increased the sensitivity of ovarian cancer cells to the growth inhibition induced by high-dose E2, but also transformed the estrogen-unresponsive SKOV3 cells from a hormone-independent to an estrogen-responsive phenotype.

### *WFDC2* knockdown promotes apoptosis induced by high-dose estrogen

Since E2 was shown to inhibit ovarian cancer cell growth, we investigated whether high-dose hormone treatment induced apoptosis in ovarian cancer cells. In this study, we performed flow cytometry and annexin V-FITC/PI staining to detect apoptosis of estrogen-responsive HO8910 cells and estrogen-unresponsive SKOV3 cells.

**Fig. 3:**
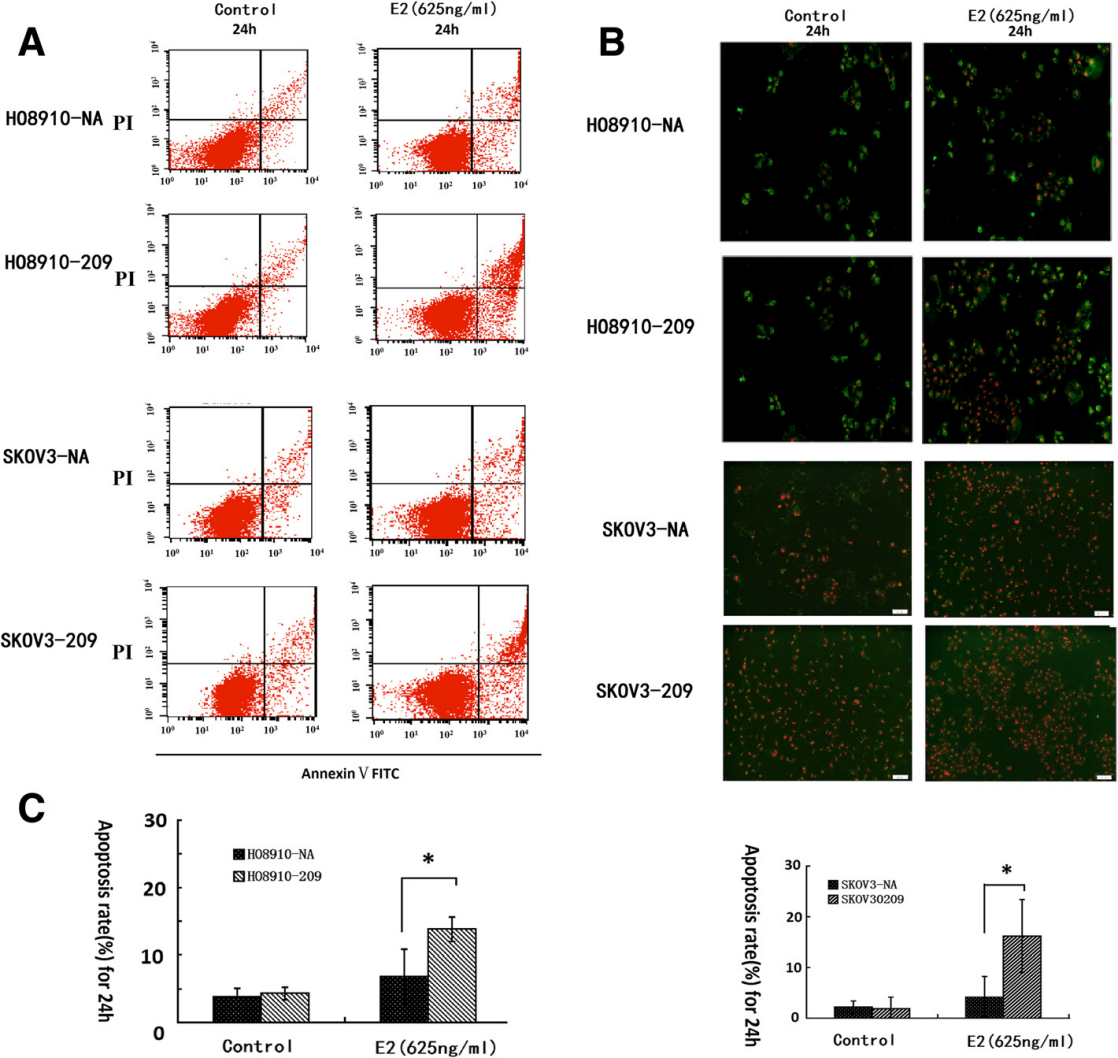
Knockdown of *WFDC2* increases cell apoptosis induced by estrogen. **a** The fraction of apoptotic cells detected using flow cytometry. **b** Apoptotic activity of 625 ng/ml E2 in *WFDC2* knockdown cells and control cells detected using Annex V-FITC/PI staining. **c** Quantification of the percentages of apoptotic cells in *WFDC2* knockdown cells and control cells treated with 625 ng/ml E2. *P < 0.05 compared with the control

The influence on cell cycle of high-dose estrogen was limited, while cell apoptosis was significantly increased by E2 in both *WFDC2*-knockdown cells and the control group, particularly in *WFDC2*-knockdown cells (Fig. 3a).

As shown in Fig. 3c, the percentage of apoptotic HO8910-209 cells [21.6 % (±6.24)] was significantly higher than that of HO8910-NA cells [12.4 % (±4.28)] when treated with E2 (*p* < 0.05). The same results were observed with SKOV3 cells (Fig. 3c). The percentage of apoptotic SKOV3-209 cells [16.2 % (±7.2)] was significantly higher than that of SKOV3-NA cells [9.6 % (±5.68)] when treated with E2 (*p* < 0.05). Thus, knockdown of *WFDC2* expression in ovarian cancer cells increased cell apoptosis induced by high-dose E2 in both estrogen-sensitive and estrogen-insensitive cells.

### Knockdown of *WFDC2* decreases expression of ERβ in HO8910 and SKOV3 cells

Given that *WFDC2* knockdown transformed a hormone-unresponsive ovarian cancer cell line in an estrogen-responsive one, we were eager to know how this would be reflected at the gene expression level. For this purpose, we studied expression of ERα and ERβ.

**Fig. 4:**
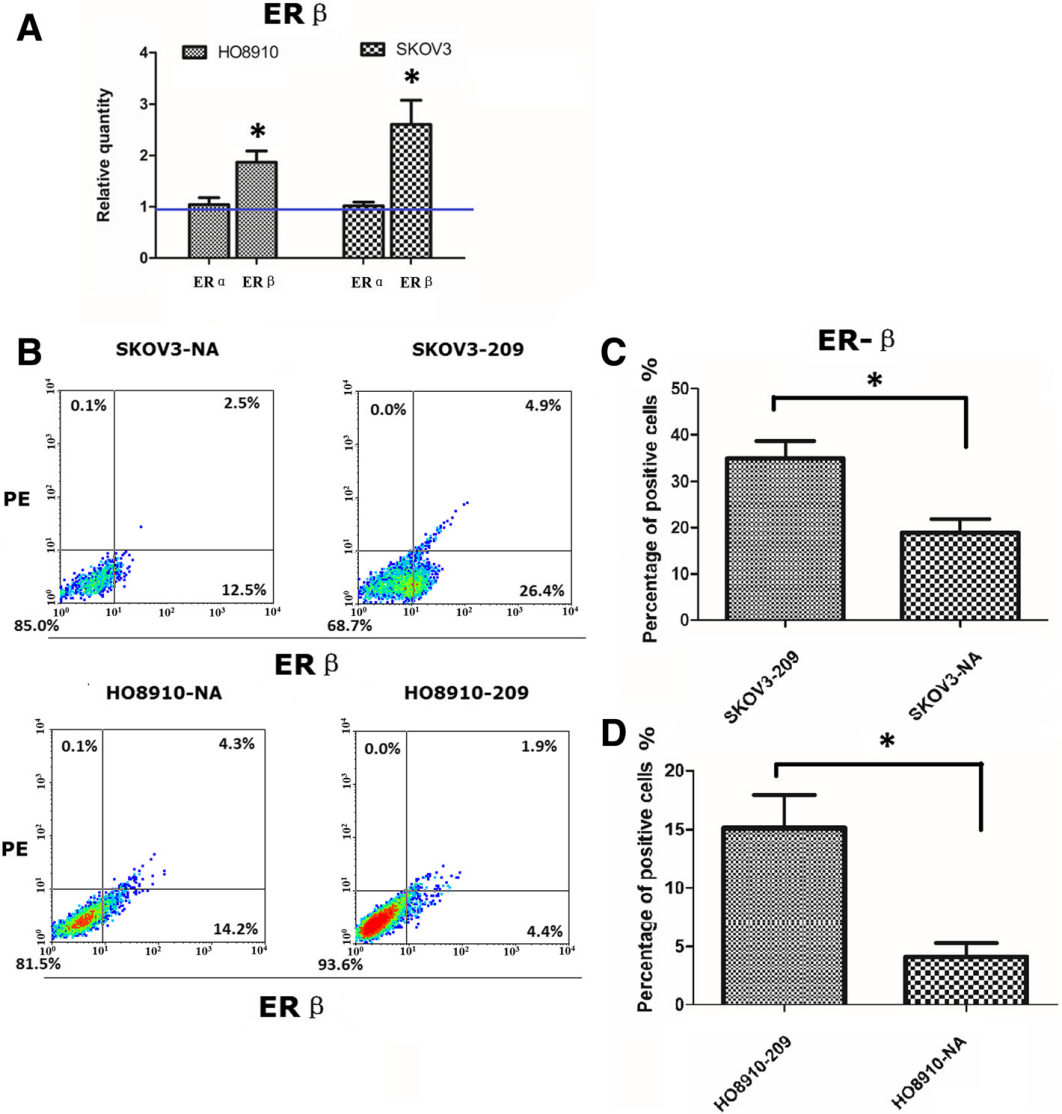
Knockdown of *WFDC2* increases expression of ERß in HO8910 and SK-OV-3 cells. **a** Normalized ERα and ERß RNA levels in *WFDC2* knockdown cells and control cells using real-time RT-PCR. The relative quantities of ERα and ERß were determined using densitometry and normalized with GAPDH. *P < 0.05 versus the control. **b** Flow cytometric graphs for ERß expression in *WFDC2* knockdown cells and control cells. **c** Quantification of the percentages of ERß-positive cells in *WFDC2* knockdown cells and control cells. *P < 0.05 versus the control

As shown in Fig. 4a, knockdown of *WFDC2* expression led to no change in the level of ERα mRNA in either HO8910 or SKOV3 cells. In contrast, expression of ERβ was strongly increased in both cell lines.

We then used flow cytometry to determine whether upregulation of ERβ expression occurred at the protein level. HO8910 cells exhibited very weak ERβ protein expression but both HO8910 and SKOV3 cells showed upregulation of ERβ expression in *WFDC2*-knockdown cell lines. The percentage of positive cells with ERβ expression was higher in *WFDC2*-knockdown cells than in the negative control cells (Fig. 4b).

### *WFDC2* regulates genes related to cell apoptosis under estrogen treatment

**Fig. 5:**
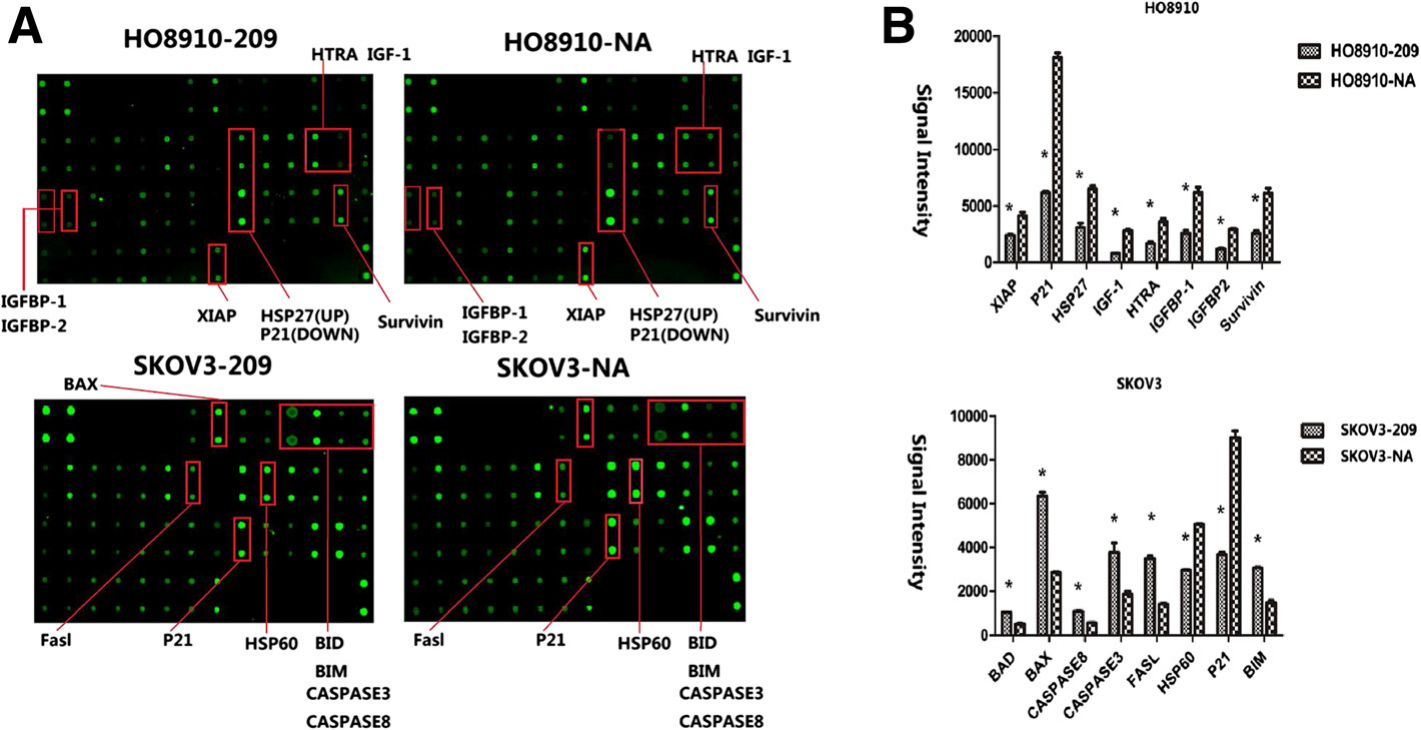
Genes related to cell apoptosis induced by E2 were modified by *WFDC2* knockdown. **a** Original figure of RayBio® Human Apoptosis Antibody array. **b** Graph of gene expression detected using the Human Apoptosis Antibody array. *P < 0.05 compared with the control

To explore the molecular mechanisms, RayBio® Human Apoptosis Antibody array was used to screen protein expression of a range of apoptosis-related genes (Fig. 5a). Figure 5b shows the protein microarray signal intensities for apoptotic-related genes in HO8910-NA and HO8910-209 cells. Notably, the microarray intensities of HSP27, IGF-1, IGFBP, P21, HTRA, survivin and XIA were more highly suppressed in HO8910-209 than in HO8910-NC cells.

In hormone-unresponsive SKOV3 cells, we observed that the microarray protein intensities of Bad (Bcl-2 antagonist of cell death), Bax (Bcl-2 associated X protein), Bim (Bcl-2 interacting mediator), caspase 3, caspase 8 and Fasl were higher, and the microarray intensities of P21 and Hsp60 were lower. Though the genes affected by *WFDC2* knockdown were not identical in estrogen-sensitive HO8910 and estrogen-insensitive SKOV3 cells, using these data, we hypothesized that *WFDC2* might represent a mechanism by which estrogen signaling induced cell apoptosis.

## Discussion

Estrogen plays a crucial role in the control of development, sexual behavior and reproductive functions. Its effects have been linked to the progression of the majority of human ovarian cancers and acts as a potent mitogen for many ovarian cancer cell lines [3, 23]. In this study, we undertook to prove the hypothesis that *WFDC2* is regulated by estrogen and that it might play a role in tumorigenesis induced by estrogen in ovarian cancer.

In this study, we demonstrated that high-dose E2 induced the upregulation of *WFDC2* gene expression in estrogen-sensitive HO8910 cells, while no induction effect was observed in estrogen-insensitive SKOV3 cells. However, the estrogen selective inhibitor TAM could not block estrogen-induced *WFDC2* expression. These results provide evidence of a positive relationship between *WFDC2* expression and estrogen action; however, the regulatory effect might not be through the ERα pathway only, which will require further experiments to prove definitively.

This study also provides the first evidence for a functional role of the *WFDC2* gene in the estrogen-dependent proliferation of ovarian cancer cells. It is generally considered that the proliferative effects of estrogen in cell culture occur at picomolar or nanomolar concentrations, typical levels in serum [1]. However, the ovary is an estrogen secretion organ, and thus micromolar concentrations of estrogen occur within the ovary and play a role in cell biology or transformation of normal ovarian surface epithelium and ovarian cancer cells, which has been largely overlooked [24, 25]. Proliferation in response to physiological concentrations of E2 has been reported in cultured ovarian cancer cells expressing ER, but currently there is no direct evidence in support of the hypothesis that high E2 levels present within the microenvironment of the ovary following ovulation contribute to the induction of ovarian cancer. Although physiological concentrations of estrogen can stimulate breast cancer cell proliferation, high-dose estrogens cause regression of some ER-positive human breast tumors.

In this study, we observed significant inhibition of cell growth induced by high-dose estrogen in estrogen-sensitive HO8910 cells, and a slight inhibition of cell growth in estrogen-insensitive SKOV3 cells. The suppression of the endogenous *WFDC2* in ovarian cancer cells not only inhibited cell growth, but also significantly strengthened the response of estrogen-insensitive SKOV3 cells to estrogen. These data suggest the involvement of *WFDC2* in estrogen signaling and in estrogen-responsiveness of ovarian cancer cells. We also found that *WFDC2* knockdown could affect the hormone-dependent proliferation not only in HO8910-sensitive but also in SKOV3-insensitive cells. *WFDC2* knockdown decreased proliferation of SKOV3 ovarian cancer cells and, more strikingly, switched on a strong estrogen response in this estrogen-unresponsive cell line.

This transformation of ovarian cancer cells from hormone-independent to an estrogen-responsive phenotype by knockdown of a single gene was further validated in proliferation experiments combining E2 with TAM. As shown in the results, *WFDC2*-knockdown increased the sensitivity of cells to TAM not only in estrogen-sensitive, but also in estrogen-insensitive cells. The suppression of the endogenous *WFDC2* in ovarian cancer cells was more likely suppressed by TAM. Thus, the specific role of *WFDC2* in cell growth in the microenvironment with high-dose estrogen may be associated with ovarian epithelial cancer cell growth and transformation, and may also be involved in TAM resistance.

Previously, we demonstrated that the expression of *WFDC2* promoted cell proliferation as well as G1/S transition, while stimulating cyclin D1 expression [12]. Cyclin D1 has been confirmed as one of the estrogen target genes, and confers the mitogenic role of estrogen [26]. These results led us to postulate that *WFDC2* participated in estrogen-dependent proliferation through regulating expression of cell cycle checkpoint proteins. While, no previous evidence has been presented, *WFDC2* has been shown to be associated with cell apoptosis [12]. We further analyzed whether apoptosis, regulated by estrogen, could be affected by *WFDC2* expression. Using FACS analysis, we showed that high concentrations of estrogen could significantly increase the level of cell apoptosis, and that loss of *WFDC2* expression resulted in a significant increase in the rate of apoptosis under high-dose estrogen both in estrogen-sensitive and insensitive cells. Our results demonstrated that pharmacological concentrations of estrogen induced apoptosis in human ovarian cancer cells that involved *WFDC2* expression.

ER has been considered to be an important regulator of estrogen-sensitive cell behavior. The response of neurons to estrogen depends on the ER subtype expressed in the cell. ERα is known to stimulate proliferation in response to estrogens by increasing expression of genes connected with cell cycle progression like cyclin D1 or growth factors, and by downregulating antiproliferative and proapoptotic genes [26, 27]. Lokich et al. demonstrated that *WFDC2* interacted with ERα, and that *WFDC2* overexpression resulted in ERα downregulation in ovarian cancer cells [19]. In our study, we did not observe upregulation of ERα in HO8910-209 cells; indeed, expression of ERα was almost unchanged by *WFDC2* knockdown. While *WFDC2* knockdown resulted in upregulation of ERβ in both HO8910 and SKOV3 cells. ERβ has been described to act as an antagonist of ERα in certain settings and to act as a tumor suppressor with proapoptotic and antiproliferative properties. Furthermore, loss of ERβ in ovarian epithelial cells has been linked to tumorigenesis and has been shown to increase proliferation of ovarian cancer cells [6, 28]. A clear upregulation in the level of ERβ was noted in *WFDC2*-knockdown cells, which could partially explain why high-dose estrogen caused more apoptosis in those cells. Thus, we hypothesize that upregulation of ERβ caused by suppression of *WFDC2* is part of the mechanism underlying the decreased growth of these cells.

Because of these results, we further studied the correlation between *WFDC2* and a series of genes that were related to cell proliferation and apoptosis using an apoptosis antibody array. In estrogen-sensitive HO8910 cells, insulin-like growth factor-1 (IGF-1) and insulin-like growth factor binding protein-1 and 2 (IGFBP-1 and IGFBP-2), which have been considered to be estrogen-responsive genes and related to the malignancy and metastasis of ovarian cancer [29–31], were downregulated in *WFDC2*-knockdown cells in the protein array. *WFDC2* knockdown has also been shown to decrease HSP27 expression, which has also been identified as an estrogen-binding protein, playing a complex role in cell proliferation, migration and differentiation of estrogen-responsive tumors [32, 33]. X-linked inhibitor of apoptosis protein (XIAP) and survivin, apoptotic suppressors [34–36], were also found to be downregulated in *WFDC2*-knockdown cells. These results are consistent with our previous hypothesis and explain the role of *WFDC2* in estrogen-dependent cell proliferation and apoptosis induced by estrogen in ovarian cancer cells. However, not all the results were in line with our expectations. We expected, that the expression of tumor suppressor genes P21 [37] and HTRA [38, 39] should be increased by *WFDC2* knockdown. But in fact, expression of these two genes in *WFDC2*-209 cells was significantly downregulated.

The results we observed in SKOV3 cells were also not entirely consistent with what we observed in the HO8910 cells. There was no evidence to confirm the relationship between *WFDC2* and the insulin-like growth factor pathway, and we did not observe any change in IGFBP-A, IGF-1, HSP27 or XIAP in SKOV3 cells, although the data were of interest. It is well known that Bcl-2 family proteins are key regulators of apoptosis [40, 41], in particular, where involvement of the mitochondrial apoptotic pathway is concerned. In estrogen-insensitive SKOV3 cells, proapoptotic genes belonging to the Bcl2 family, such as Bad, Bax, and Bim were upregulated by *WFDC2* knockdown. These data led us to suppose that *WFDC2* could take part in cell apoptosis through the mitochondrial apoptotic pathway. Accordingly, proapoptotic genes, Fasl, Hsp60, caspase 3 and caspase 8 were also upregulated in *WFDC2*-knockdown cells, which also revealed the proapoptotic effects of *WFDC2* knockdown. Additionally, the tumor suppressor gene P21 was downregulated in *WFDC2*-knockdown SKOV3-209 cells, and as seen in HO8910-209 cells. The contradictory results suggest that *WFDC2* might be involved in a complex apoptotic net that will need further study to unravel.

The inconsistent results obtained using SKOV3 and HO8910 cells suggested that *WFDC2* regulated cell apoptosis through different pathways in estrogen-sensitive and estrogen-insensitive cells. The focus of our upcoming research, therefore, will be to gain a better understanding of the relationship between *WFDC2* and the insulin-like growth factor pathway to ascertain the role of *WFDC2* on the physiological and pathological behavior of ovarian cancer. However, it was the downregulation of the tumor suppressor genes P21 and HTRA, which led us to question the pathophysiological role of *WFDC2* in tumor initiation and progression. Understanding the detailed molecular mechanisms of action of *WFDC2* in cell apoptosis induced with high-dose E2 will require further study.

## Conclusion

In summary, understanding how estrogen is involved in ovarian cancer development and progression is important for determining strategies and targets for ovarian cancer prevention and treatment. For the first time, we, have identified *WFDC2* as an estrogen target gene that might be involved in estrogen-induced physiological and pathological events. We also proved that *WFDC2* affected tumor growth in the presence of high-dose estrogen through gene expression regulation, although further work on the specific mechanism is needed. Additional studies of *WFDC2* could provide significant information as to its potential pathophysiological actions in ovarian cancer progression and might lead to novel strategies for tumor treatment in patients treated with anti-estrogens.

## Additional file

Additional file 1: Figure S1. Expression of *WFDC2* in SKOV3 cells silences clonal lines. (A) Western blot analysis of expression of *WFDC2* and GAPDH in SKOV3 cells*.* Normalized *WFDC2* protein levels in the shRNA-transfected (SKOV3-309, SKOV3-209), mock-transfected SKOV3-NA and control SKOV3 cells. The relative quantities of *WFDC2* protein were determined by densitometry and normalized to *GAPDH.* **P* < 0.05 compared with SKOV3-NA; #*P* < 0.05 compared with SKOV3. (B) Western blot analysis of expression of *WFDC2* and GAPDH in HO8910 cells*.* Normalized *WFDC2* protein levels in shRNA-transfected and mock-transfected NA cells. Relative quantities of *WFDC2* protein were determined using densitometry and normalized to GAPDH*.* **P* < 0.05 compared with HO8910-NA; (DOC 293 kb)

## Abbreviations

*WFDC2*: WAP four-disulfide core domain 2
ERα/ß: estrogen receptor alpha/beta
EMT: epithelial-mesenchymal transition
IHC: immunohistochemistry
qPCR: quantitative real-time PCR
NC: negative control
HRT: hormone replacement therapy
IGFBP: insulin-like growth factor binding protein
TFF: trefoil factor
FP: follicular
OP: ovulatory
E2: 17-ß-estradiol
SERM: selective ER modulator
TAM: tamoxifen
