# Supplementary material for: WAP four-disulfide core domain protein 2 gene(WFDC2) is a target of estrogen in ovarian cancer cells
Source: J Ovarian Res. 2016 Feb 29;9:10. doi: 10.1186/s13048-015-0210-y (PMC4770698; doi:10.1186/s13048-015-0210-y)
Supplement: Additional file 1: Figure S1. — Expression of WFDC2 in SKOV3 cells silences clonal lines. (A) Western blot analysis of expression of WFDC2 and GAPDH in SKOV3 cells. Normalized WFDC2 protein levels in the shRNA-transfected (SKOV3-309, SKOV3-209), mock-transfected SKOV3-NA and control SKOV3 cells. The relative quantities of WFDC2 protein were determined by densitometry and normalized to GAPDH. *P < 0.05 compared with SKOV3-NA; #P < 0.05 compared with SKOV3. (B) Western blot analysis of expression of WFDC2 and GAPDH in HO8910 cells. Normalized WFDC2 protein levels in shRNA-transfected and mock-transfected NA cells. Relative quantities of WFDC2 protein were determined using densitometry and normalized to GAPDH. *P < 0.05 compared with HO8910-NA; (DOC 293 kb) [file 13048_2015_210_MOESM1_ESM.doc]

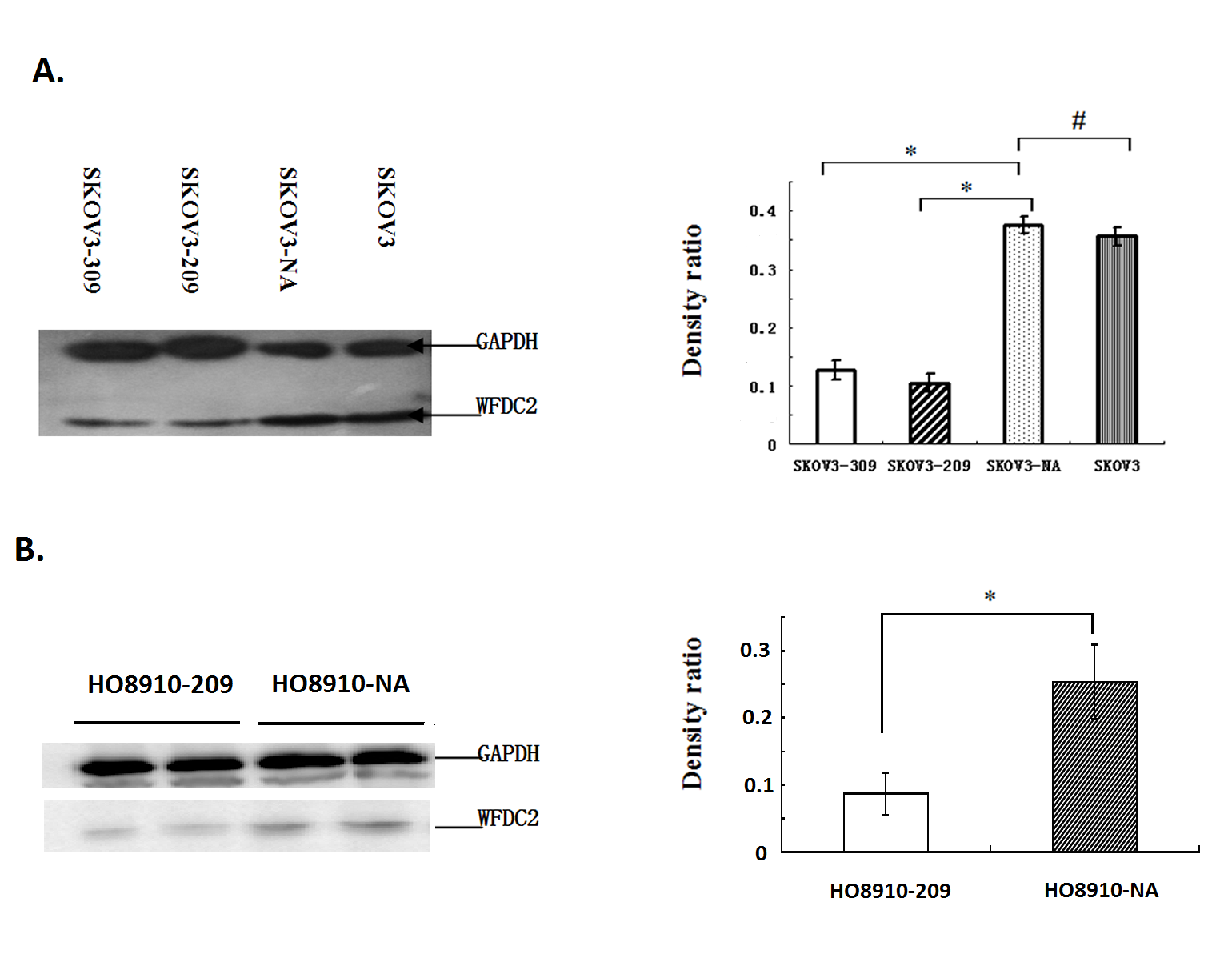


**Figure. 1** **Expression of WFDC2** **in WFDC2 silences Clonal Lines.**

**（A）**Western blot analysis of expression of *WFDC2* and *GAPDH* in SKOV3 cells*.* Normalized *WFDC2* protein levels in the *shRNA*-transfectant, mock-transfectant NA and control. The relative quantities of *WFDC2* protein were determined by densitometry and normalized by using *GAPDH.* *P <0.05 compared to SKOV3-NA; #P <0.05 compared to SKOV3. **（B）**Western blot analysis of expression of *WFDC2* and *GAPDH* in HO8910 cells*.* Normalized *WFDC2* protein levels in the *shRNA*-transfectant, mock-transfectant NA. The relative quantities of *WFDC2* protein were determined by densitometry and normalized by using *GAPDH.* *P <0.05 compared to HO8910-NA;
